# Drug Reaction with Eosinophilia and Systemic Symptoms (DRESS) caused by niraparib: a novel antineoplastic agent

**DOI:** 10.1186/s13223-025-00959-2

**Published:** 2025-10-02

**Authors:** Irene Vázquez-Barrera, Alba Juárez-Guerrero, Cristina Cuevas-Bravo, Patricia Rojas Perez-Ezquerra, Blanca Noguerado-Mellado

**Affiliations:** Allergy Department, Hospital General Universitario Gregorio Marañón, Madrid, Spain

**Keywords:** DRESS syndrome, Hypersensitivity reaction, Drug allergy, Niraparib, Rash

## Abstract

**Background:**

Drug Reaction with Eosinophilia and Systemic Symptoms (DRESS) is a rare but potentially life-threatening hypersensitivity reaction characterized by skin rash, fever, lymphadenopathy, hematologic abnormalities, and organ involvement. Niraparib, a poly (ADP-ribose) polymerase (PARP) inhibitor, is used to treat ovarian, fallopian tube, or primary peritoneal cancer. Although niraparib is associated with cutaneous toxicities, no severe cutaneous adverse reactions (SCARs) have been reported until now.

**Case presentation:**

We present a case of DRESS syndrome in a 73-year-old woman with high-grade serous ovarian cancer treated with niraparib. After 20 days of therapy, she developed a widespread maculopapular rash. Despite discontinuation of niraparib and treatment with corticosteroids, she exhibited pruritus, facial edema, lymphadenopathy, eosinophilia, and impaired liver and renal function. A RegiSCAR score of 6 confirmed the diagnosis of DRESS. Patch testing to niraparib 1% in DMSO was positive when performed nine weeks after DRESS resolution.

**Conclusions:**

This is the first reported case of DRESS by hypersensitivity due to niraparib. This case highlights the importance of recognizing DRESS as a potential adverse reaction to niraparib and the efficacy of early corticosteroid intervention. Further research is needed to understand and mitigate the risk.

## Background

Drug Reaction with Eosinophilia and Systemic Symptoms (DRESS) is a rare, life-threatening hypersensitivity reaction. DRESS syndrome is characterized by generalized skin rash, fever, and lymphadenopathy, along with involvement of at least one internal organ and hematological abnormalities, primarily eosinophilia and lymphocytosis. The European Registry of Serious Cutaneous Adverse Reactions (RegiSCAR) group has established diagnostic criteria for this condition [1].

Niraparib, a poly (ADP-ribose) polymerase (PARP) inhibitor, was approved by the Food and Drug Administration in 2017. It works by blocking the enzymes responsible for DNA repair, thus inducing cytotoxicity in cancer cells. Niraparib is primarily used to treat epithelial ovarian, fallopian tube, or primary peritoneal cancer. It has been associated with cutaneous toxicities such as rash, hyperhidrosis, photosensitivity reactions and peripheral edema [2]. No severe cutaneous adverse reaction (SCAR) has been reported.

We present a case of DRESS syndrome in a patient following treatment with Niraparib, with sensitization confirmed through skin testing. Informed consent was obtained from the patient to publish the case report along with all accompanying visual elements.

## Case presentation

**Fig. 1:**
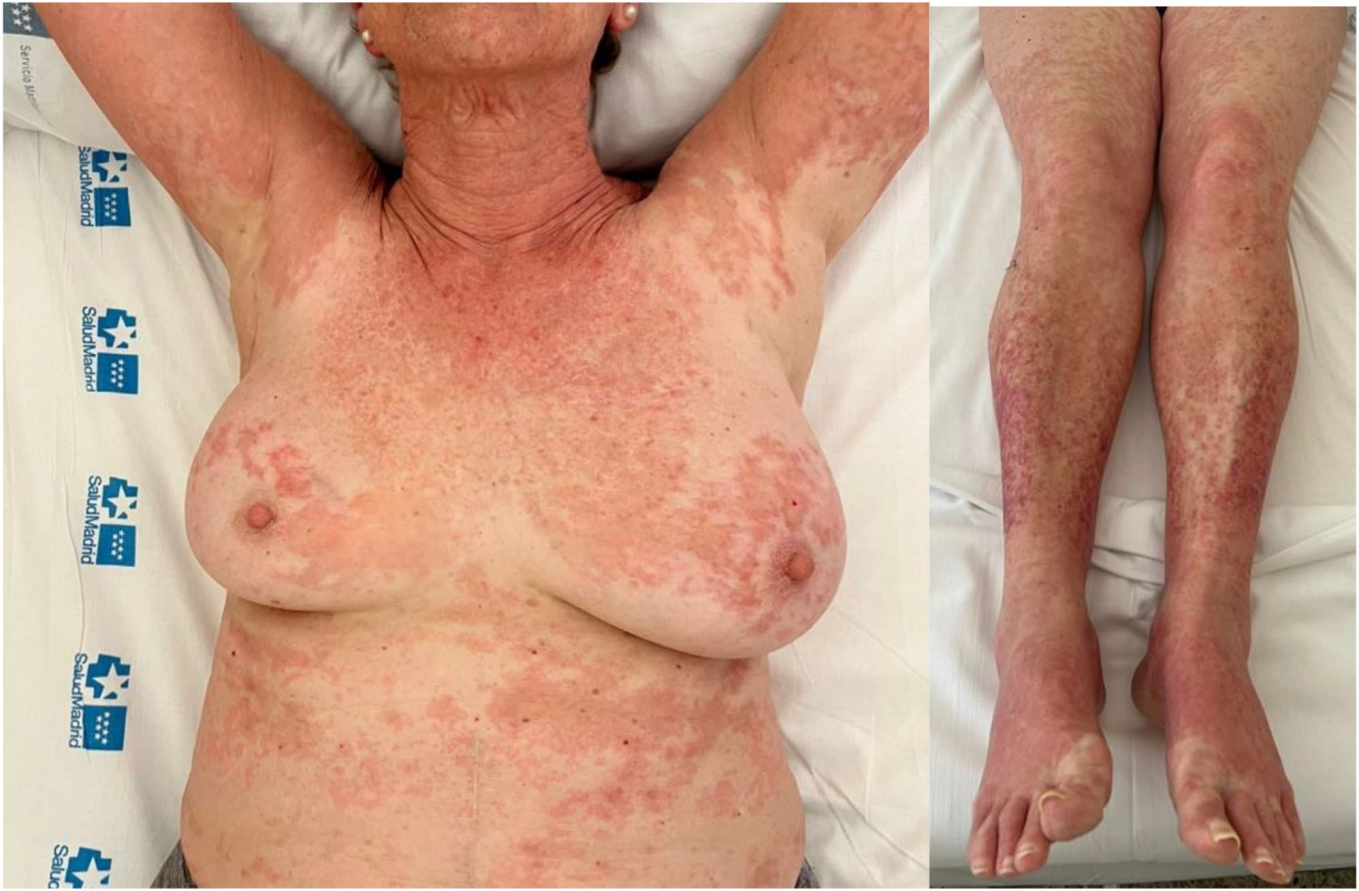
Coalescing maculopapular rash on her face, trunk, and extremities

A 73-year-old woman with high-grade serous ovarian cancer and peritoneal dissemination presented to the emergency room with a generalized rash. She had no personal history of allergy. Her medical history included a femoropopliteal deep vein thrombosis in the left lower limb, caused by ovarian cancer. Twenty days prior, she began a maintenance therapy regimen of 200 mg per day of niraparib. In addition, the patient was taking letrozole, calcifediol, and apixaban. Upon examination, she exhibited a coalescing maculopapular rash on her face, trunk, and extremities, including the palms (Fig. 1).

Upon presentation, treatment with 20 mg of intramuscular methylprednisolone was administered, and oral methylprednisolone 16 mg/day was prescribed. Niraparib was discontinued. Despite this treatment, twelve days later, she developed severe pruritus, facial edema and cervical adenopathies. Blood test revealed eosinophilia (3200 cells/µL), worsening renal function (creatinine from 47,74 µmol/L to 86,64 µmol/L) and abnormal liver profile (alanine aminotransferase (ALT) from 19 U/L to 154 U/L, aspartate aminotransferase (AST) from 19 U/L to 74 U/L and gamma-glutamyl transpeptidase (GGT) from 22 U/L to 45 U/L). Serologies were negative for HIV, HBV, HCV, EBV, CMV, syphilis and parvovirus. ANA test was negative.

She was admitted to the hospital and started on 40,8 mg/day (0.8 mg/kg/day) of methylprednisolone IV. This dose was maintained for 5 days, followed by a reduction to 25,5 mg/day (0.5 mg/kg/day) for 4 days, and then transitioned to 32 mg/day of oral methylprednisolone for an additional 3 days before discharge. Her skin lesions and pruritus improved by the fifth day of hospitalization, the eosinophilia count and renal and liver function normalized after twelve days of hospitalization, allowing for discharge.

On discharge, 26 more days of systemic corticosteroids were prescribed to complete the tapering. She later tolerated letrozole, calcifediol, and apixaban, and it is now part of her regular treatment.

A punch biopsy was performed 15 days after the onset of skin lesions, revealing dermatopathologic features suggestive of a drug reaction. Histologically, the sample shows a skin layer lined by epidermis of usual thickness and an orthokeratotic chorneal layer, exhibiting vacuolar degeneration of the basal layer associated with a moderate superficial and lichenoid mixed perivascular inflammatory infiltrate composed of lymphocytes, eosinophils, macrophages, and scattered plasma cells. No microabscesses, micropustules, or granulomatous inflammation were observed.

According to the RegiSCAR, she had a score of 6, confirming a definite diagnosis of DRESS (Table 1).

**Table 1:** Results of the RegiSCAR scoring system

Items	Patient’s result	Score
Fever ≥ 38.5ºC	No	-1
Enlarged lymph nodes (≥ 2 sites, > 1 cm)	No	0
Eosinophilia 700–1499 ≥ 1500	Yes	2
Atypical lymphocytes	No	0
Skin rash > 50% of body surface area	Yes	1
At least 2 of: edema, infiltration, purpura, scaling	Yes	1
Biopsy suggestive of DRESS	Yes	0
Internal organ involved 1 ≥2	Yes	2
Resolution in > 15 days	Yes	0
3 tests of the following test were performed and all were negative: HAV, HBV, HCV, Mycoplasma, Chlamydia, ANA, blood culture	Yes	1

**Fig. 2:**
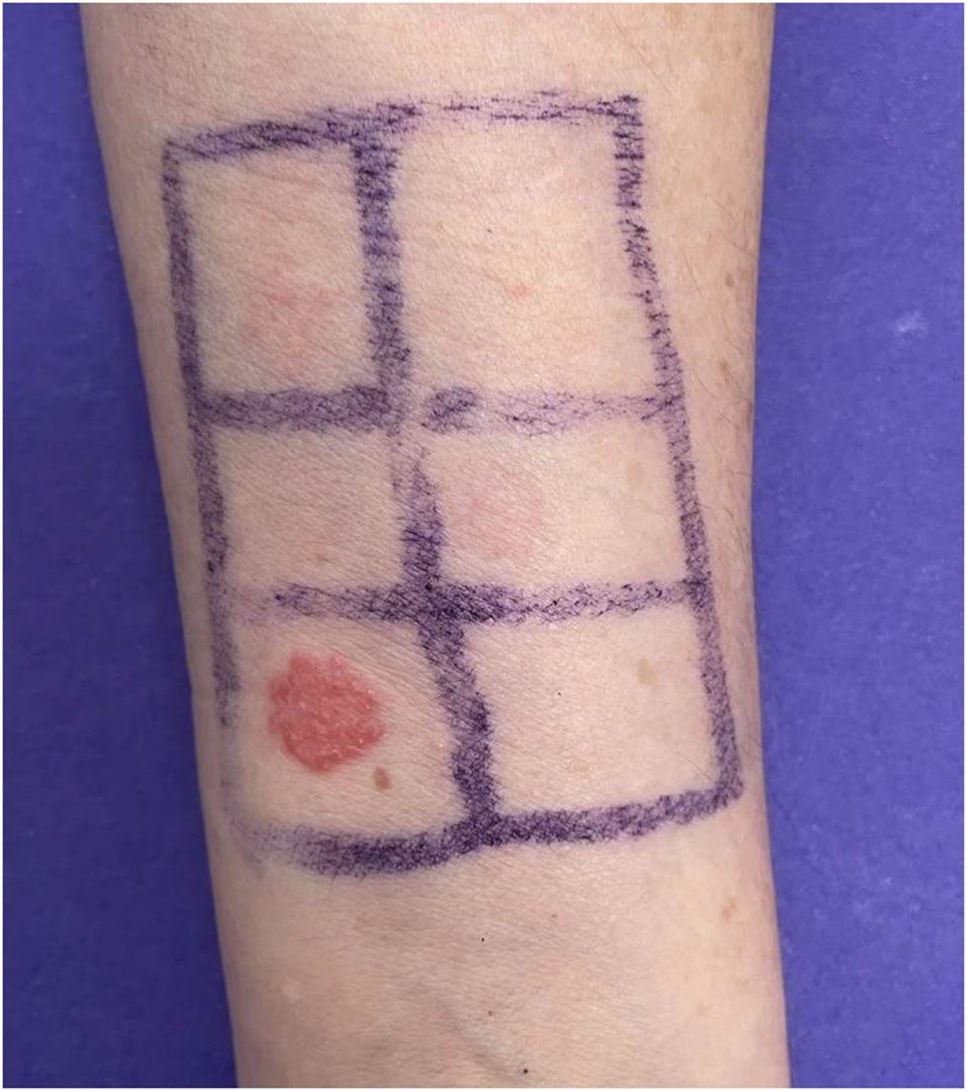
Patch tests. Image shows significant positivity to the culprit drug (Niraparib) at D4

The patient was referred to the Allergy Department. Patch test (PT) were performed nine weeks after the patient was discharged from the hospital, and she had not taken corticosteroids for a week. The tests included olaparib 1% in dimethyl sulfoxide (DMSO), olaparib 1% in aqua, niraparib 1% in DMSO, and niraparib 1% in aqua. Readings were made on day 2 (D2) and day 4 (D4). The PT were positive for niraparib 1% in DMSO at D2 (++) and D4 (+++). Control patch test with DMSO was negative (Fig. 2).

The patient presented with fluctuating alterations in liver function tests despite having discontinued niraparib treatment months earlier, with transaminase elevations that improved with corticosteroid therapy and a normal liver biopsy. Additionally, four months after discontinuing niraparib, the patient developed bilateral panuveitis. Persistent liver involvement secondary to DRESS should be considered as a possibility, as well as the potential for DRESS to induce autoimmunity. For these reasons, provocation testing with olaparib was not performed, despite negative patch test results. The patient is currently on letrozole treatment.

## Discussion and conclusions

We highlight the importance of patch test in the etiological diagnosis of DRESS. We used niraparib 1% in DMSO for the patch test, as it is a vehicle that enhances the penetration of substances into the skin. This is evident because niraparib at 1% in aqua tested negative, while in DMSO it tested positive. Control patch test with DMSO was negative. A possible limitation of our study is that we did not perform a patch test with niraparib 1% in DMSO on a patient tolerant to niraparib. Therefore, we cannot confirm whether niraparib 1% in DMSO is an irritant or if the result is truly positive, although, based on the patient’s history, we can reasonably speculate that it is.

To the best of our knowledge, this is the first case report of DRESS by hypersensitivity due to niraparib confirmed by positive PT. This case underscores the importance of recognizing DRESS syndrome as a potential adverse reaction to niraparib. The early intervention with corticosteroids proved effective, and the patient’s condition improved following discontinuation of niraparib. Future research should focus on understanding the mechanisms behind niraparib-induced DRESS and developing strategies to mitigate this risk in cancer patients requiring PARP inhibitors.

## Data Availability

No datasets were generated or analysed during the current study.

## Abbreviations

DRESS: Drug Reaction with Eosinophilia and Systemic Symptoms
PARP: Poly (ADP–ribose) Polymerase
SCAR: Severe Cutaneous Adverse Reaction
RegiSCAR: European Registry of Serious Cutaneous Adverse Reactions
PT: Patch Test
DMSO: Dimethyl Sulfoxide
